# Tolerance and Persistence to Drugs: A Main Challenge in the Fight Against *Mycobacterium tuberculosis*

**DOI:** 10.3389/fmicb.2020.01924

**Published:** 2020-08-26

**Authors:** Francesca Boldrin, Roberta Provvedi, Laura Cioetto Mazzabò, Greta Segafreddo, Riccardo Manganelli

**Affiliations:** ^1^Department of Molecular Medicine, University of Padova, Padova, Italy; ^2^Department of Biology, University of Padova, Padova, Italy

**Keywords:** mycobacteria, tuberculosis, persistence, tolerance, stringent response

## Abstract

The treatment of tuberculosis is extremely long. One of the reasons why *Mycobacterium tuberculosis* elimination from the organism takes so long is that in particular environmental conditions it can become tolerant to drugs and/or develop persisters able to survive killing even from very high drug concentrations. Tolerance develops in response to a harsh environment exposure encountered by bacteria during infection, mainly due to the action of the immune system, whereas persistence results from the presence of heterogeneous bacterial populations with different degrees of drug sensitivity, and can be induced by exposure to stress conditions. Here, we review the actual knowledge on the stress response mechanisms enacted by *M. tuberculosis* during infection, which leads to increased drug tolerance or development of a highly drug-resistant subpopulation.

## Introduction

**Figure 1:**
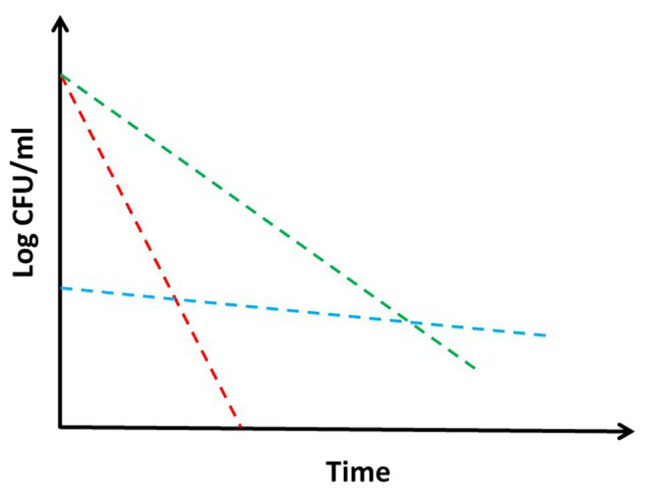
Theoretical drug killing curves of different bacterial populations: sensitive (red), persistant (blue), and tolerant (green). When a persistant subpopulation is present in a bacterial culture, the shape of the killing curve appears as bimodal. Explanation in the text.

Tolerance and persistence: two words often used as synonyms, but indicating deeply different concepts and biological phenomena (Kussell et al., 2005). It is current knowledge that bacteria are particularly difficult to be killed by drugs in some situations, even though they are sensitive to them, causing as a consequence relapse of the disease (Meylan et al., 2018). This is particularly true for bacteria as *Mycobacterium tuberculosis*, which needs to be treated with complex drug cocktails for extremely long time in order to be defeated, therefore increasing the risk to reduce compliance and induce deleterious side effects of drugs (Kaul et al., 2019). Consequently, understanding the mechanisms underlying these phenomena is of major interest to predict drug efficacy and to design treatment strategies in order to target bacteria, which are hardly killed by standard drug regimen. Despite the large amount of literature regarding mechanisms of tolerance and persistence in bacteria in general and in mycobacteria in particular, often the two terms are not clearly defined, with some authors even proposing that distinction between the two is contrived (PMID: 28082974). Moreover, the experiments are rarely designed to really distinguish between the two of them (Brauner et al., 2016). Additionally, the definition of these two terms is sometimes confused or diverse among different schools of thought. Usually, tolerance is defined as an increase in the survival times of bacterial cells toward a bactericidal drug without any change of the minimal inhibitory concentration (MIC), and it is applied to the entire bacterial population upon exposure to a peculiar and often stressful environment (Figure 1; Brauner et al., 2016; Meylan et al., 2018). This does not involve the selection of resistant mutants, but is usually due to a reduction of metabolism that consequently leads to growth rate slowdown upon encountering specific environmental conditions. On the other hand, persistence is characterized by the onset of bacterial subpopulations, which are extremely resistant to drug concentrations several times higher than the MIC, with the rest of the population remaining sensitive (Kussell et al., 2005; Brauner et al., 2016). Consequently, persistence is characterized by a killing curve with a bimodal trend: a first part with a steepest slope, during which the sensitive (or tolerant) population is killed by the drug, and a second part, which is flat or with a reduced slope, representing the behavior of the persistent subpopulation (Figure 1). Like tolerance, also persistence is not due to genetic modifications, since persistent bacteria, once isolated, do not show any increase in drug susceptibility (Brauner et al., 2016; Harms et al., 2016; Trastoy et al., 2018).

Some authors consider as persisters only those cells that are not dividing at all, defined as dormant, whereas othersinclude in the persisters also the minor subpopulation of dividing cells showing a higher drug resistance or drug tolerance compared to the rest of the bacterial population (Brauner et al., 2016; Kim and Wood, 2017). Practically, tolerance can be considered as a consequence of the individual response of a single cell to stress, in order to survive longer in a dangerous situation. Since all cells in a culture are exposed to the same environment, all of them react in the same way, and the entire population becomes tolerant. However, persistence can be considered as a social behavior used by a bacterial community to guarantee the survival of at least a minority of its members toward incoming stress conditions, through the preventive development of heterogeneous subpopulations with different physiologic characteristics. Such a heterogeneity can be obtained by deterministic or stochastic mechanisms. In the latter case, stochastic fluctuations can be amplified by stressful environmental signals and drive to differential gene expression leading to heterogeneity.

The experiments to discriminate among these phenomena can be quite complex and time-consuming, especially when working with a slow-growing microorganism such as *M. tuberculosis*, as they require time to kill curves to be performed under different physiologic conditions in order to examine their kinetics, or the use of microfluidic cultures and time-lapse microscopy (Brauner et al., 2016). However, most of the published data, at least in the mycobacterial field, are based on limited observations, so that often the discrimination between tolerance and persistence is difficult (Antonova et al., 2018).

In this paper, we review the actual knowledge about the mechanisms of drug tolerance and persistence to drugs in *M. tuberculosis*, underlining their vast complexity.

## Stimulation of Respiration Reduces Drug Persistence

A clear evidence that persistence to drugs in *M. tuberculosis* is due to the presence of different cellular subpopulations was given by Jain et al. (2016). These authors characterized the transcriptional profile of *M. tuberculosis* persisters survived after a 4-days exposure to an isoniazid (INH) concentration 20 times higher than the MIC. Their results highlighted two different sets of differentially expressed genes. One set included genes that were typically differentially expressed after 4 h of exposure to INH, such as *iniBAC*, encoding an efflux pump known to expel INH from the cytoplasm. A second set, included genes not previously associated to INH treatment. Among the latter, there were several downregulated genes encoding proteins involved in cell division and energy metabolism, which suggested a population of slow growing bacteria, and upregulated genes involved in stress response, such as *dnaK*, *hsp*, or *clpB*. Using dual-reporter mycobacteriophages, the authors showed that several of the stress response genes induced in persisters, were also induced in a bacterial subpopulation of logarithmic-phase cultures not treated with any drug, whose size increased upon exposure of the culture to different environmental stress. This data suggested that a subpopulation of “likely persister” cells is already present before exposing a culture to drugs, and its size can be increased upon exposure to environmental stress. Finally, using time-lapse microscopy, they could also observe that survival to INH was more likely to occur in cells in which one of the persisters-associated genes (*dnaK*) was induced (Jain et al., 2016). Interestingly, when bacterial cultures were treated with small thiols, such as cysteine or DTT, the size of the *dnaK*-expressing “likely persister” population was dramatically reduced, consequently also the number of bacteria able to survive INH or rifampicin decreased. As these compounds are able to shift the menaquinol/menaquinone balance toward a reduced state, which stimulates respiration, it was hypothesized that they may drive persisters to a more metabolically active state, which restores their sensitivity to drugs (Vilcheze et al., 2017).

## Cellular Heterogeneity by Asymmetric Division: A Way to Develop Heterogeneous Populations With Different Drug Susceptibility

**Figure 2:**
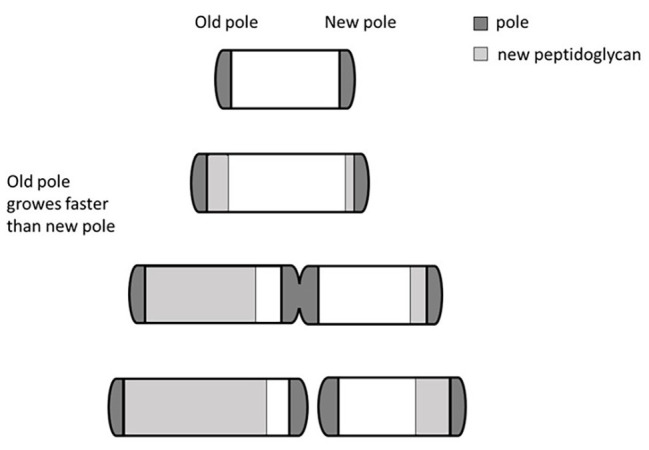
Bipolar growth of mycobacteria. Rapid cell elongation occurs at the older pole (here arbitrarily shown on the left) that grows faster than the newly formed pole. This asymmetric division generates a heterogeneous population in both size and elongation rate. Different individuals of the heterogeneous population can have different drugs sensitivity.

One mechanism used by mycobacteria to generate cell-to-cell deterministic heterogeneity is based on their peculiar cellular growth and division. Unlike other rod-shaped bacilli that divide by originating identical cells, and whose elongation follows a spiral shape along the main body of the microorganism, mycobacteria miss the ruler that allows accommodation of the division site at the center of the cell (Singh et al., 2013a). Consequently, they elongate from their poles, with the older pole (inherited from the mother cell) growing faster than the newly formed pole (Figure 2). As a result, after each division cycle, the cell inheriting the growing pole (accelerator) elongates faster and becomes larger than the other one (alternator), which needs to regenerate a new growing pole. Therefore, such a division mechanism generates a heterogeneous population both in size and elongation rate (Aldridge et al., 2012; Kieser and Rubin, 2014). The molecular mechanism underneath this phenomenon has been described, with *LamA* (formerly Mmps3), a member of the divisome, inhibiting growth at nascent new poles (Rego et al., 2017). Several evidences indicated that *Mycobacterium smegmatis* cells of different size, generated by asymmetric division, had different sensitivity to drugs: accelerator cells were shown to be more susceptible than alternators to drugs targeting the cell wall biosynthesis such as cycloserin, meropenem, and INH, however, they were less susceptible to rifampicin (Aldridge et al., 2012; Richardson et al., 2016; Vijay et al., 2017a), suggesting that asymmetric cell division can contribute to the formation bacterial subpopulations more tolerant to drugs. Interestingly, deletion of *lamA* in *M. tuberculosis* leads to the loss of single-cell heterogeneity and faster killing by vancomycin and rifampicin (Rego et al., 2017). Finally, *M. tuberculosis* cells grown in stressful conditions, such as oxidative stress or iron deficiency, or recovered from sputum or infected macrophages, were shown to have increased cell-size heterogeneity, suggesting that asymmetric cell division might play a role in the variable outcome of treatment of *M. tuberculosis* (Vijay et al., 2017b).

## Persistence to Isoniazid Due to Katg Pulses

In their interesting work, Wakamoto et al. (2013) characterized the behavior of *M. smegmatis* exposed to 10X MIC of INH. In this condition, the bacterial killing curve was characterized by a first short stage in which bacteria continued to divide, a second stage of rapid bacterial death, and a third stage in which the number of alive bacteria remained stable, suggesting the presence of non-replicating persisters. However, using time-lapse microscopy in association with a microfluidic device, the authors showed that the latter phase was characterized by a dynamic state of the culture in which the rate of cell division and cell death were similar; challenging the hypothesis that persistence is always due to non-growing cells. As INH is a prodrug that needs to be activated by the catalase KatG, the authors hypothesized that persisters might express its gene at a lower level. When they analyzed KatG expression at a single cell level, they found that it was expressed in short pulses followed by a rapid degradation. Statistical analyses determined that, depending on the frequency of the pulses, cells were more or less likely to reach the intracellular KatG concentration required for INH activation, driving consequently to heterogeneity. KatG pulsing, and consequent capacity to survive or not in the presence of INH, was correlated between sibling cells, but was not an inheritable trait, suggesting the presence of stochastic variation due to an epigenetic mechanism. Unfortunately, these data were entirely obtained in *M. smegmatis*, often used as a surrogate of *M. tuberculosis* for its low pathogenicity and fast growth, and still await to be confirmed in *M. tuberculosis*.

## Persistence to Rifampicin: A Multifactorial Phenomenon

Beyond the “classical” non-dividing persisters, other subpopulations able to survive rifampicin treatment have been described. A first example is represented by those generated by the effect of mistranslation of the *rpoB* gene, encoding the β-subunit of RNA polymerase, which is the target of rifampicin. Mistranslation is a common feature of living cells and can be due to errors either of the ribosome or the transfer RNA (tRNA) synthase. It has been shown that mycobacteria can replace glutamate with glutamine and aspartate with asparagine (Javid et al., 2014). Resistance to rifampicin is usually due to *rpoB* gene mutations in a specific region, which lead to transitions either from glutamate to glutamine or aspartate to asparagine. Consequently, it was hypothesized that mistranslation of these amino acids could result in the presence of a fraction of RNA polymerase molecules resistant to rifampicin in the cell. Accordingly, an increase in the mistranslation rate in *M. smegmatis* resulted in an increased bacterial survival upon rifampicin exposure (Javid et al., 2014).

Moreover, a strain with a ribosomal mutation that conferred higher ribosome fidelity, exhibited a decreased number of persisters able to survive rifampicin treatment. Since mistranslation occurs more frequently in stressful environments, it is conceivable that the number of bacterial cells with an amount of rifampicin-resistant RNA polymerase exceeding the threshold required to surviving in the presence of rifampicin, increases in stressed bacterial populations.

**Figure 3:**
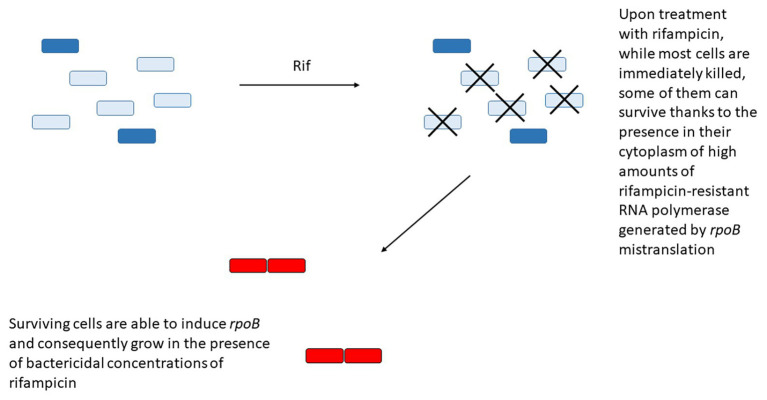
Some bacteria can survive rifampicin treatment due to the presence in their cytoplasm of high concentration of rifampicin-resistant RNA polymerase (dark blue) due to *rpoB* mistranslation. These cells will have the opportunity to overexpress the wild type RNA polymerase (red) from its secondary promoter and gain transient resistance to the drug.

Another example is represented by the paradoxical induction of *rpoB* expression in a subpopulation of cells upon treatment with rifampicin. The region upstream *rpoB* contains two SigA-dependant promoters, with the first promoter responsible for the basal level of expression. When the RNA polymerase activity is inhibited by low doses of rifampicin or fidaxomiycin, inhibition of transcription from the first promoter allows the induction of the second promoter, leading to RpoB accumulation and transient resistance to the drug (Zhu et al., 2018). In an interesting working model, Zhu et al. (2018) proposed that upon treatment with rifampicin, while most cells are immediately killed, some of them can survive thanks to the presence in their cytoplasm of high amounts of rifampicin-resistant RNA polymerase generated by *rpoB* mistranslation. These cells are then able to induce *rpoB* from its second promoter and consequently grow in the presence of bactericidal concentrations of rifampicin (Figure 3). Even if most of the data were obtained in *M. smegmatis*, and still await to be confirmed in *M. tuberculosis*, the presence of a rifampicin-inducible promoter upstream *rpoB* in *Mycobacterium bovis* BCG, whose *rpoB* region is identical to that of *M. tuberculosis* suggests that this mechanism is present also in this species (Zhu et al., 2018).

## Role of Toxin-Antitoxin Systems in Development Of Persisters in *M. Tuberculosis*

The genomes of mycobacteria belonging to the *M. tuberculosis* complex encode 77 toxin-antitoxin (TA) modules, a disproportionate number compared to the five modules encoded by non-tubercular mycobacteria such as *M. smegmatis* (5 TAs), *Mycobacterium marinum* (4 TAs), or *Mycobacterium ulcerans* (3 TAs; Slayden et al., 2018). Most of the *M. tuberculosis* TA modules belong to type II, characterized by a toxin with endoribonuclease activity and an antitoxin that binds the toxin to neutralize its action. Specific environmental stimuli induce antitoxin degradation, allowing the toxin to exert its action on its specific target, such as the ribosome, specific tRNAs, or messenger RNAs (mRNAs; Slayden et al., 2018; Barth et al., 2019) with a consequent slow-down of metabolism and arrest of cellular division. TA modules can be stochastically induced, allowing the presence of non-growing (persister) subpopulations either in the absence of stress or upon stimulation by different environmental cues leading to an increase in the size of such subpopulations. The presence of so many TA systems in *M. tuberculosis* suggests that they may have a very important role in its life-style. It has been hypothesized that these systems are responsible not only for *M. tuberculosis* persistence to drugs, but also for the detection and integration of the environmental stimuli encountered by the bacteria during infection, which direct them toward the development and maintenance of the dormant state typical of latent tuberculosis (Slayden et al., 2018). The role of *M. tuberculosis* TA systems in persistence to drugs has been characterized for TAs belonging to the RelBE and MazEF subgroups. In both cases, induction of the toxin component in the absence of its cognate antitoxin leads to bacteriostasis and lower susceptibility to the killing action of bactericidal drugs. Moreover, *M. tuberculosis* mutants missing one or more of these TA systems were shown to be killed by drugs at higher frequency than their wild type parental strain (Singh et al., 2010; Tiwari et al., 2015). Interestingly, each TA system was able to provide increased survival to a specific drug, or set of drugs, suggesting a specific and diversified role of the different TA systems (Singh et al., 2010; Tiwari et al., 2015). Unfortunately, even if these data strongly suggest the involvement of TA systems in persistence development, neither time-lapse microscopy to follow the fate of single cells or time to kill experiments at different time points were performed, therefore the precise mechanism undergoing this phenomenon still awaits full clarification.

## Mycobacterial Biofilms, a Role in Tolerance and Persistence

When *M. tuberculosis* is grown unshaken, it forms a thick biofilm pellicle at the air-medium interface, which requires iron and zinc and is stimulated by CO_2_ and low oxygen tension (Ojha et al., 2008). A peculiar characteristic that differentiates these biofilms from planktonic grown cells is the presence of abundant extractable free mycolic acids including methoxy and in lesser extent, keto mycolates (Ojha et al., 2008). Interestingly, Sambandan et al. (2013) demonstrated that keto mycolic acids, but not methoxy mycolic acids, are essential for *M. tuberculosis* biofilm formation. Bacterial biofilms are usually enriched in cells that survive drug treatment both for their inaccessibility to the drugs and for their slow growth/metabolism. Accordingly, mycobacterial biofilms were shown to be extremely tolerant to INH, and contain high number of persisters surviving high concentrations of rifampicin (Ojha et al., 2008). Similar findings were obtained when *M. tuberculosis* cells were grown as microbial communities attached to untreated well surface or to wells covered by extracellular matrix. In this case, dispersion of the bacilli with a detergent or with DNase I restored drug susceptibility (Ackart et al., 2014). A Tn-seq screening for *M. tuberculosis* mutants unable to form biofilms revealed that several genes required for biofilm formation were also involved in stress and drug tolerance. It is possible that the harsh environment found in the biofilm, characterized by nutrients and oxygen depletion and mechanical stresses, selects a bacterial population intrinsically tolerant to exogenous stresses, including drug exposure, therefore increasing the frequency of persisters (Richards et al., 2019).

In another study, using a different biofilm model, the *M. tuberculosis* persister population exhibited a shift of trehalose metabolism. This was characterized by the diversion of this molecule from the synthesis of cell surface components as trehalose monomycolate and trehalose dimycolate, to the synthesis of central carbon metabolism intermediates to maintain the essential functions, thus leading to the development of dormant bacteria (Lee et al., 2019).

*M. tuberculosis* forms large clusters resembling biofilms both extracellularly, in necrotic lesions, and intracellularly, suggesting that biofilm formation could be clinically relevant and contribute with the induction of tolerance and/or persistence to decrease the effect of drug treatment (Basaraba and Ojha, 2017).

## Role of Sigma Factors in Tolerance and Persistence

Sigma factors are small proteins able to bind the RNA polymerase holoenzyme to confer promoter specificity. Usually bacterial genomes encode one essential sigma factor, responsible for transcription of essential housekeeping genes, and a variable number of alternative sigma factors whose expression and/or activity is activated in response to specific environmental stimuli (Paget, 2015). *M. tuberculosis* genome encodes 13 sigma factors, making this bacterium the obligate pathogen with the highest ratio of sigma factors/Mb (Rodrigue et al., 2006). One of the best characterized *M. tuberculosis* sigma factor is SigE, an extra cellular function (ECF) sigma factor essential for the response to surface stress, low pH, and oxidative stress (Manganelli, 2014). SigE is also responsible for the transcription of the genes encoding the alternative sigma factor: SigB, the pleiotropic regulator ClgR and the two component system MprAB (Manganelli, 2014). Moreover, it is involved in the development of the stringent response (see below). SigE, whose activity is regulated by a very complex regulatory network, has been proposed as one of the major switches for dormancy (Balazsi et al., 2008; Dona et al., 2008; Manganelli and Provvedi, 2010; Tiwari et al., 2010).

In 2017, we showed that a *M. tuberculosis sigE* mutant was more susceptible than its parental strain to several drugs, including INH, rifampin, streptomycin, gentamycin, vancomycin, and ethambutol, whereas a *sigB* mutant was more sensitive only to INH and ethambutol which both target mycolic acids biosynthesis (Pisu et al., 2017). Moreover, we showed that persisters able to escape INH and streptomycin killing occurred less frequently in both mutants compared to the wild type, while those escaping killing by vancomycin appeared less frequently than in the wild type only in the *sigE* mutant. Interestingly, we also found that the bacteriostatic drug ethambutol was bactericidal for both mutants (Pisu et al., 2017). Even if the molecular basis of these findings awaits further studies, these data underline the role of stress response pathways in the development of tolerance and persistence to drugs in *M. tuberculosis* and open the possibility of targeting sigma factors to find alternative strategies to shorten tuberculosis treatment.

## Role of Stringent Response in Tolerance to Drugs

Stringent response is an integrated response to several different types of stresses including C, Fe, and phosphate limitation, which reallocates cellular resources that allow the development of a semi-dormant state helping the cell to survive in hostile environments (Gaca et al., 2015). In *Escherichia coli* such a response is triggered by RelA/SpoT homolog (RSH) proteins, which synthesize the alarmone (p)ppGpp in response to starvation (Hauryliuk et al., 2015). The increased concentration of (p)ppGpp has several effects on bacterial physiology including (i) inhibition of several translation factors, DNA primase, and GTP biosynthesis, (ii) transcriptional repression of genes encoding ribosomal RNA (rRNA), and (iii) induction of genes required for nutrient acquisition, amino acid biosynthesis, and stress survival (Hauryliuk et al., 2015). Some of these effects are mediated by the increased concentration of polyphosphate (poly P), e.g., activation of TA systems by inhibition of the Lon protease. It is not surprising that such a response in *E. coli* was shown to increase the amount of persisters surviving drug treatment (Hauryliuk et al., 2015).

**Figure 4:**
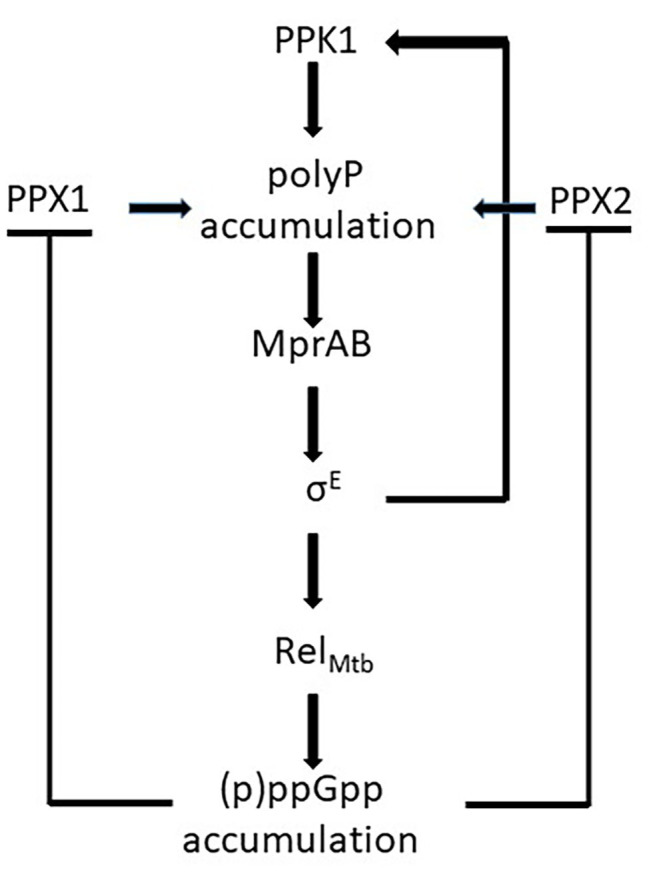
Schematic representation of the effects of (p)ppGpp accumulation in *Mycobacterium tuberculosis*: the inhibition of the exopolyphosphatases PPX1 and PPX2 leads to poly P accumulation and to the activation of the *mprAB-sigE-rel_mtb_* signaling pathway. Poly P accumulation is enhanced by the induction of *ppk1*.

Unlike *proteobacteria*, which encode two RSH enzymes with different specific functions, mycobacteria encode only one of them: RelA*_Mtb_*, with a (p)ppGpp hydrolyzing domain and a (p)ppGpp synthase domain. In stressful conditions, the catalytic domain is activated leading to (p)ppGpp accumulation in the cytoplasm. As a consequence, activities of the exopolyphosphatases PPX1 and PPX2 are inhibited leading to poly P accumulation (Choi et al., 2012; Chuang et al., 2015, 2016) and activation of the *MprAB-SigE-Rel_mtb_* signaling pathway that includes the induction of the poly P kinase PPK1, further increasing poly P accumulation (Manganelli, 2007; Sureka et al., 2007, 2008; Figure 4). SigE positively regulates both *ppk1* and *rel_mtb_* transcription, and is expected to have a central role in the development of the stringent response (Sanyal et al., 2013). This hypothesis is corroborated by the deeply different transcriptional response to phosphate starvation observed in a *M. tuberculosis sigE* mutant compared to its parental strain (manuscript in preparation).

Poly P accumulation in *M. tuberculosis* causes a metabolic downshift and alteration of cell wall permeability, resulting in antibiotic tolerance (Chuang et al., 2015, 2016). This is supported by the finding that a *M. tuberculosis* mutant missing PPX1 is associated with elevated intracellular poly P and tolerance to INH (Thayil et al., 2011), whereas a mutant devoid of PPK1 is associated to low levels of poly P and increased drug susceptibility (Singh et al., 2013b). In a recent work, Dutta et al. (2019) demonstrated that a *M. tuberculosis rel_mtb_* null mutant was unable to accumulate poly P and down-modulate metabolism to decrease growth rate in a condition of nutrient starvation. While the INH minimal bactericidal concentration (MBC) of the mutant strain and its parental strain were equal in rich media, during nutrient starvation the wild type strain MBC increased 512-fold, whereas that of the mutant strain remained equal, suggesting that the stringent response activation was responsible for tolerance to this drug. Interestingly, enhanced susceptibility to INH of the *rel_mtb_* null mutant was also demonstrated during the chronic phase of infection in BALB/c mouse lungs (Dutta et al., 2019). The finding that (p)ppGpp-mediated tolerance to INH was detectable only upon nutrient starvation, well explains the contrasting data obtained by Bhaskar et al. (2018), who did not show any tolerance to INH in different *M. smegmatis* mutants missing *rel_mtb_*, *lon*, *ppk1*, *ppk2*, or both *ppx1* and *ppx2*, as these authors run their experiments in cultures growing exponentially in rich medium, a condition not activating the stringent response.

## Mechanism of Tolerance to Bedaquiline

Bedaquiline represents a new class of antitubercular drugs whose mechanism of action implies the inhibition of the F_1_F_0_-ATP synthase (Cholo et al., 2017). In an interesting study, Peterson et al. (2016) modeled the transcriptional response of *M. tuberculosis* to this drug during the first 96 h of exposure, before the bactericidal effect of bedaquiline was evident. From the analyses of the data, they identified two previously uncharacterized transcriptional regulators: Rv0324 and Rv0880, whose regulons were either upregulated in response to bedaquiline (Rv0324), or predicted to be essential in the presence of the drug (Rv0880). While most of the genes regulated by Rv0880 belong to the functional category “cell wall and cell processes” (Lew et al., 2013), Rv0324 regulon is enriched in genes belonging to the functional category “virulence, detoxification, and adaptation” including the *mce3* operon, which was recently hypothesized to be involved in bedaquiline tolerance (Yang et al., 2019). Mutants devoid of Rv0324 or Rv0880 were killed by bedaquiline faster than their wild type parental strain, despite their MIC was the same: the typical signature of tolerance. When exposed to other antitubercular drugs, the mutants showed the same phenotype as the wild type, suggesting that Rv0880 and Rv0324 govern a gene network able to develop a state of tolerance specific for bedaquiline.

## Ribosome Hibernation in Response to Zinc Depletion and Tolerance to Aminoglycosides

When *M. tuberculosis* is exposed to a low zinc environment, the zinc uptake regulator (Zur) activates the expression of several genes involved in the adaptation to zinc starvation, including alternate ribosomal proteins missing the zinc binding motif CxxC (C-; Maciag et al., 2007). Beyond allowing the bacteria to recycle the zinc previously immobilized in ribosomes, such a condition permits the assembly of ribosomes with different characteristics and helps them to survive. Zinc-free ribosomes are targeted by a mycobacterial-specific Y protein homolog (MPY), which confers structural stability to the ribosome and competes with mRNA, tRNA and elongation factors, leading to minimal level of transcription and consequently a metabolic slowdown, which helps growth-arrested bacteria to survive until zinc is newly available (Li et al., 2018). Another effect of MPY binding to the ribosome is its interference with aminoglycosides binding. Indeed, under zinc starvation conditions, a *M. smegmatis* strain whose operon encoding the alternate C-ribosomal proteins was deleted more susceptible to streptomycin and kanamycin than its parental wild type strain.

Using a reporter plasmid to measure the expression of the *M. tuberculosis* operon encoding C-ribosomal proteins during mice infection, it was shown that during the acute infection about 14% of the bacteria expressed the reporter, whereas, during chronic infection, this value increased to 40% and was reduced when zinc was added to the drinking water (Li et al., 2018). This is in line with the increase of zinc concentration observed in the phagosomal environment during acute infection (Botella et al., 2011), as well as with its decrease in necrotizing lesions, typical of the chronic phase of the infection, due to its chelation by calprotectin secreted by neutrophils in response to activation of the adaptive immunity (Corbin et al., 2008). Taken together, these data suggest that during chronic infection, ribosome remodeling induced by zinc depletion contributes to aminoglycoside tolerance due to increased stability of the ribosome, reduced metabolism, and lower accessibility of the aminoglycosides binding site. While the induction of the alternate ribosomal proteins known to form C-ribosomes in low zinc environment was shown in *M. tuberculosis*, the role of C-ribosomes in metabolic slowdown and aminoglycoside tolerance has been until now shown only in *M. smegmatis*.

## Role of Isocitrate Lyases in Oxidative Stress-Induced Tolerance

Induction of intracellular oxidative stress is a very well-known consequence of treatment with several antibacterial drugs and has been hypothesized to be one of the major killing mechanisms of bactericidal drugs, regardless of their mechanism of action (Kohanski et al., 2007).

Using a metabolomic approach, Nandakumar et al. (2014) found that treatment of *M. tuberculosis* with sublethal concentrations of INH, rifampicin or streptomycin induced a common subset of metabolites belonging to the tricarboxylic acid cycle (TCA) cycle, glyoxylate metabolism, and amino acid biosynthetic pathway. In particular, the authors focused on the TCA cycle, since it is involved in oxidative damage leading to the production of hydroxyl radicals following antibiotic treatment (Kohanski et al., 2007). They found that antibiotic treatment induced a metabolic response causing the activation of its bifunctional isocitrate lyases (ICL) and methylisocitrate lyases. As a consequence, the glyoxylate shunt was intensified, whereas the activity of the reductive arm of the TCA cycle was reduced, suggesting an adapting respiratory slowdown to counteract the increase of reactive oxygen intermediates (ROI) induced by antibiotic treatment. Accordingly, a strain missing ICL showed induction of several ROI responsive genes, suggesting a role of ICL in counteracting endogenous oxidative stress and was more sensitive to INH, rifampicin, and streptomycin. Moreover, this phenotype was complemented by the thiol-based antioxidant thiourea (Nandakumar et al., 2014).

Taken together, these data suggest that activation of ICLs as a reaction to antibacterials treatment represents a response to counteract oxidative stress induced by antibacterials, thus representing an intrinsic mechanism of drug tolerance (Nandakumar et al., 2014).

## Triacylglicerol Accumulation as a Trigger for Bacterial Hibernation and Drug Tolerance

When *M. tuberculosis* is exposed to stresses such as low iron, low pH, or low oxygen, it accumulates large amounts of intracellular triacylglicerol (TAG) droplets. This is mainly due to the induction of *tgs1*, encoding a well-characterized TAG synthase which redirects carbon fluxes from intermediary metabolic pathways to TAG biosynthesis, thus decreasing metabolism and growth rate (Baek et al., 2011; Maurya et al., 2018). Indeed, *tgs1* null mutants were shown to be unable to reduce their growth rate under low iron, low pH, or low oxygen conditions (Baek et al., 2011). Beyond reducing growth rate, TAG accumulation has been proposed to play a fundamental role as carbon storage allowing rapid restoration of metabolic activity during resuscitation (Daniel et al., 2004), and to maintain redox homeostasis in conditions of low respiration (Leistikow et al., 2010).

The direct link between TAG accumulation and phenotypic drug tolerance is well-demonstrated by the fact that *M. tuberculosis* cultures subjected to growth-limiting conditions able to induce intracellular TAG accumulation and became more tolerant to several drugs only if they have a functional *tgs1* gene (Deb et al., 2009; Baek et al., 2011).

Some interesting observation suggested that TAG accumulation is linked to the development of caseum-induced tolerance (Sarathy et al., 2018). During infection, the interaction between *M. tuberculosis* and host factors induces remodeling of tissue that generates typical lesions of chronic inflammation known as granulomas. With the progression of the infection, the center of granulomas undergoes a process of remodeling implying the accumulation of necrotic material with formation of cheese-like necrotic material known as caseum, that eventually may liquefy leading to the process of cavitation. Liquefied caseum that reaches an adjacent airway stimulates the cough reflex facilitating bacterial dissemination (Marakalala et al., 2016). Granulomas caseous centers represent a pro-inflammatory environment characterized by anoxia and the presence of several proteins and lipids deriving from cellular necrosis, as well as reactive oxygen species, pro-inflammatory eicosanoids, and antimicrobial peptides (Marakalala et al., 2016; Seto et al., 2019). Sarathy et al. (2018) showed that *M. tuberculosis* residing in caseum is not able to replicate and become highly tolerant to several first‐ and second-line anti tubercular drugs following a trend similar to that observed in *in vitro* models of non-replicative *M. tuberculosis* based on hypoxia or starvation. Even if the mechanism leading to caseum-induced tolerance is not fully identified, the accumulation of TAG droplets in caseum-residing bacilli suggests that in these environment bacteria, probably due to anoxia, ROS, and antimicrobial peptides induce *tgs1*, which leads to their hibernation by reducing their metabolism and growth rate. Bacteria found in sputum, which mainly derive by necrotic caseus material, are rich in TAG droplets and are highly tolerant to antibacterial drugs, suggesting that tolerance acquired in caseum is clinically relevant (Dhillon et al., 2014; Barr et al., 2016; Turapov et al., 2016).

## *glpK* Phase Variation as a Rapidly Reversible Genetic Mechanism of Genetic Tolerance

GlpK is an enzyme required for glycerol catabolism *via* the lower glycolytic pathway and is essential for growth in media with glycerol as the sole carbon source (Bellerose et al., 2019). A *M. tuberculosis* mutant missing this gene was recently found to be tolerant to several drugs when bacteria were grown on glycerol (Bellerose et al., 2019). Interestingly, two independent groups recently showed that *glpK* is subjected to phase variation due to frequent and reversible frameshift mutations in a homopolymeric 7G sequence present at the 5' half of its open reading frame. As a consequence of this phase variation mechanism, some clinical strains produce subpopulations forming small colonies with a smooth phenotype, with heritable and rapidly revertible tolerance to several drugs. Interestingly, these variants accumulate during drug treatment, suggesting their relevance in treatment failure and relapse (Bellerose et al., 2019; Safi et al., 2019).

## Macrophage-Induced Tolerance

*M. tuberculosis* genome encodes a large amount of efflux pump systems compared to its genome size, whose role in intrinsic and acquired drug resistance is well-known (Milano et al., 2009; Ramon-Garcia et al., 2009, 2012; Kanji et al., 2019). However, recent evidences suggest the involvement of some efflux pumps also in the development of drug tolerance during infection. Adams et al. (2011) using a zebrafish larval model found that a subpopulation of intracellular *M. marinum* was able to escape killing from INH. Following this finding, the same authors demonstrated that macrophage residence induced tolerance to INH, rifampicin, and moxifloxacin also in *M. tuberculosis* and this tolerance was associated with a replicating bacterial population. Since several efflux pumps are known to be induced during macrophage infection, the authors hypothesized that drug efflux could have a role in intracellular drug tolerance. To validate their hypothesis they treated infected resting macrophages with drug efflux inhibitors, which indeed reduced drug tolerance demonstrating a role of efflux pumps in macrophage-induced tolerance (Adams et al., 2011). A recent study shed light on the mechanism by which resting macrophage residency induces drug tolerance in *M. tuberculosis*: phagosomal acidification was found to modify redox physiology of *M. tuberculosis* generating heterogeneous populations of replicating bacteria characterized by different mycothiol redox potential (E_MSH_). The E_MSH_-reduced population was found to be enriched in acidic compartments compared to the E_MSH_-basal population and highly tolerant to several antibiotics (Bhaskar et al., 2014; Mishra et al., 2019). Transcriptional profiling of the E_MSH_-reduced population, revealed the induction of several genes involved in drug efflux pumps production, in biosynthesis of iron-sulfur clusters, in modulation of bacterial response to drugs (Kohanski et al., 2007), or in the production of hydrogen sulfide, also known to protect bacteria from oxidative stress and antibiotics (Shatalin et al., 2011).

Taken together, these data strongly suggest that low phagosomal pH-induced modification of E_MSH_ is directly responsible of induction of drug tolerance in resting macrophages. This was confirmed by the fact that treatment of infected mice with the antimalaria drug chloroquine, which deacidifies the phagosomal compartment, counteracts drug tolerance, and relapses of the disease (Mishra et al., 2019).

While *M. tuberculosis* is able to grow without any restriction in resting macrophages, activated macrophages are able to reduce its replication due to the imposition of a more severe and stressful environment. Single cell-dynamics studies revealed that exposure to nutrient starvation, intracellular replication, and growth in mice organs, increased bacterial phenotypic heterogeneity allowing the appearance of non-growing metabolically active bacterial subpopulations highly tolerant to drugs. Since this phenotypic heterogeneity is abolished in mice lacking interferon-γ, it was hypothesized that the state of macrophage activation could play a role in inducing drug tolerance (Manina et al., 2015). This hypothesis was confirmed by Liu et al. (2016) comparing the sensitivity to four frontline drugs of bacteria residing in resting or activated macrophages. These authors found that, despite some overlap, the transcriptional response of *M. tuberculosis* to INH treatment was different when bacteria were grown in axenic culture or in macrophages. In particular, they found that the genes induced by INH treatment exclusively in the intracellular environment, belonged to regulons specifically induced by exposure to low pH, nutrient starvation, nitrosative or oxidative stress, and surface-damage. Among them (i) several genes belonging to the PhoP regulon, typically induced by low pH, (ii) genes belonging to the DosR regulon, known to induce dormancy in respones to nitric oxide exposure and low pH, (iii) genes regulated by SigK and SigF in response to starvation, (iv) genes regulated by the two component system MprAB and SigE in response to surface damage, and (v) genes controlled by SigH, essential for responding to oxidative stress (Liu et al., 2016). Since all of these stressful conditions are believed to be experienced by *M. tuberculosis* during macrophage infection, these data led to the hypothesis that INH exposure increased the pressure of the intramacrophage environment on bacteria by decreasing bacterial fitness. This hypothesis was corroborated by the further finding that also exposure of intracellular bacteria to rifampicin, ethambutol, or pyrazinamide resulted in similar transcriptional response, despite their different mechanisms of action (Liu et al., 2016). Finally, it was found that the transcriptional response of intracellular *M. tuberculosis* to drugs was similar to that induced by macrophage activation leading to the hypothesis that macrophage activation, imposing a stronger stress to the intracellular bacteria, promotes their drug tolerance (Liu et al., 2016). Interestingly, in this case the phenotypic heterogeneity observed in the bacterial population would not depend on intrinsic stochastic or deterministic mechanisms, but on the variety of the experienced conditions during infection (different levels of macrophage activation).

## Conclusions

The relationship of *M. tuberculosis* with its human host is extremely complex and fascinating. The different harsh environments encountered during infection stimulate bacteria to activate specific responses evolved to modulate their physiology to resist and react to stress, allowing their long-term survival and reproduction in what represents the only possible environment for them: our body. Often these complex stress responses contribute to make the bacterial population tolerant to drugs or induce the development of different bacterial subpopulations, which can be more tolerant or extremely resistant to drugs (persistence). Consequently, the mechanisms that can make *M. tuberculosis* tolerant or persistent to drugs are several and diverse, often inducing different types of tolerance and persistence, which can coexist when different stress signals are present at the same time. These results in a continuous spectrum of phenotypes that sometimes are difficult to classify without fully understanding and dissecting them, suggesting that our definitions of tolerance and persistence can be too simplistic to describe such complex and often coexisting phenomena.

## Future Perspectives

As the problem of multi-drug resistant (MDR) tuberculosis increases its impact on the cure of this disease, a deeper understanding of the complex relationship between *M. tuberculosis* responses to the environmental cues and drug efficacy, will be of major impact on the possibility to develop more effective drugs or drug regimen. In particular, it will be extremely important to validate in *M. tuberculosis* the observations obtained in *M. smegmatis* or other non-tubercular mycobacteria and to identify and target those tolerance and persistence mechanisms with major impact on treatment length or failure.

## Author Contributions

All authors wrote the manuscript and drew the figures. All authors contributed to the article and approved the submitted version.

### Conflict of Interest

The authors declare that the research was conducted in the absence of any commercial or financial relationships that could be construed as a potential conflict of interest.
